# Utilizing an electronic portal imaging device to monitor light and radiation field congruence

**DOI:** 10.1120/jacmp.v4i4.2502

**Published:** 2003-09-01

**Authors:** J. I. Prisciandaro, M. G. Herman, J. J. Kruse

**Affiliations:** ^1^ Division of Radiation Oncology Mayo Clinic 200 First Street SW Rochester Minnesota 55905

**Keywords:** electronic portal imaging device, quality assurance, light and radiation field congruence

## Abstract

A method to investigate light and radiation field congruence utilizing a commercially available amorphous silicon electronic portal imaging device (EPID) was developed. This method employed an EPID, the associated EPI software, and a diamond‐shaped template. The template was constructed from a block tray in which Sn/Pb wires, 1 mm in diameter, were embedded into a diamond shaped groove milled into the tray. The collimator jaws of the linac were aligned such that the light field fell directly on the corners of the diamond. A radiation detection algorithm within the EPI software determined the extent of the radiation field. The light and radiation field congruence was evaluated by comparing the vertexes of the diamond reference structure to the detected radiation field. In addition, the digital jaw settings were recorded and later compared to the light field detected on the films and EPIs. Three linear accelerators were tracked for a period ranging from 2–8 months. Light radiation field congruence tests with films and EPIs were comparable, yielding a difference of less than 0.6 mm, well within the allowed 2‐mm tolerance. A disparity was observed in the magnitude of the detected light field. The *X* and *Y* dimensions of the light field measured with film differed by less than or equal to 1.4 mm from the digital collimator settings, whereas the values extracted from the EPIs differed by up to 2.5 mm. Based on these findings, EPIs were found to be a quick and reliable alternative to film for qualitative and relative analyses.

PACS number(s): 87.53.Xd, 87.56.Fc, 87.53.Oq, 87.52.–g, 87.53.–j

## INTRODUCTION

As radiation oncology facilities begin to clinically implement intensity modulated radiation therapy (IMRT), the need to develop the appropriate quality assurance (QA) tests for geometric and dosimetric plan verification becomes essential. Several authors have suggested the use of electronic portal imaging devices (EPIDs) to facilitate this procedure.^1^ ^–^ ^3^ However, prior to utilizing an EPID for IMRT quality assurance, it may be prudent to first incorporate EPIDs into conventional radiation therapy QA procedures. As a first step, for this study, an EPID was utilized to monitor light and radiation field congruence. Conventionally, this type of imaged‐based QA is performed with radiographic film. However, the use of film is time‐consuming, due to film processing, and its analysis can be subjective unless the film is digitized. Although methods to assess light and radiation field congruence with an EPID have been previously explored by Luchka *et al*.^4^ and Dunscombe *et al*.,^5^ the current study was performed using an amorphous silicon detector, which has superior image resolution compared to the video‐based systems reported previously. In addition, the test tool utilized is relatively inexpensive and easy to fabricate in‐house. By substituting electronic portal images in place of film, the efficiency and the overall quality of this and other procedures can be improved. For this study, an accurate, efficient and reliable image‐based quality assurance procedure utilizing an electronic portal imaging device is discussed.

## METHODS AND MATERIALS

**Figure 1:**
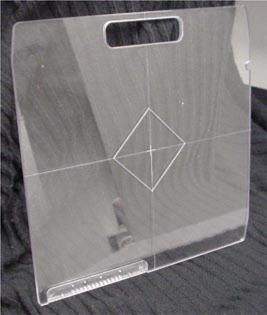
Diamond template constructed for light and radiation congruence test utilizing an EPID.

An EPID‐based light and radiation field congruence procedure was examined that utilized a commercially available EPID and the accompanying software, PortalVision™ 6.1. (Varian aS500, PortalVision 6.1, Varian Medical Systems, Palo Alto, CA). All tests were performed with a gantry and collimator angle of 0°. To define the light field, a diamond‐shaped structure was embedded within a block tray. The diamond template was created by milling a square 6.8×6.8 cm^2^ in dimension, rotated by 45°, onto the surface of a block tray (see Fig. 1). The central axis was denoted with a 1‐cm crosshair milled into the center of the tray. The grooves were milled to a depth of 1 mm. Radio‐opaque wires, consisting of a Sn/Pb alloy, 1‐mm in diameter, were pressed within these grooves. The block tray was mounted in the head of the gantry and projected a diamond structure at isocenter with a vertex‐to‐vertex distance of approximately 15 cm. The EPID was positioned 160 cm from the source, providing ~0.5 mm pixel pitch at isocenter. Each asymmetric jaw of the linac was independently adjusted to intercept a vertex of the diamond. The digital setting for the *X* and *Y* jaws were recorded and later compared to the size of the detected light field. The field was irradiated with a dose of 12 MU and electronic portal images were acquired for low‐energy (6 MV) and high‐energy (10 or 18 MV) x‐ray beams. Maintaining the jaw settings, gantry and collimator angle, a sheet of Kodak X‐Omat TL film was positioned 100 cm from the source over 5 cm of solid water backscatter material on the treatment couch. Although the diamond template was mounted in the block tray, the light field was determined by scribing the projected light field directly on the film jacket, as is done in standard practice. One centimeter of solid water build up was placed over the film and the field was irradiated with a 6X beam several minutes after the EPIs were acquired with a dose of 12 MU. A second film was acquired with a high‐energy x‐ray beam (10 or 18 MV). For each measurement, the digital collimator settings were recorded and later compared with the detected light field on the films and EPIs.

**Figure 2:**
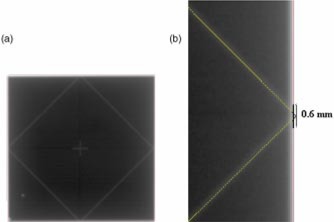
(Color) (a) An example of a portal image acquired with the diamond template mounted in the head of the gantry. To determine the light and radiation field congruence, a reference structure is overlaid on the EPI. (b) The difference between the vertex of the reference structure and the radiation field, as defined by the EPI software, yields the light radiation congruence.

The electronic portal images were analyzed with the associated EPI software. To analyze each image, a previously acquired image was stored as a reference. A diamond structure was drawn over the shadow cast by the block tray. The reference structure was aligned such that each of its sides overlaid the center of the diamond‐shaped image. Latter EPIs were compared to the reference image by overlaying the corresponding reference structure over the center of the diamond on the newly acquired EPI (see Fig. 2). The vertices of the reference structure represent the edge of the light field. The field edge detection algorithm available within the analysis package^6^ delineated the radiation field for the EPIs. The light and radiation field congruence was determined by measuring the distance between each vertex of the reference structure and the detected radiation field [see Fig. 2(b)]. The evaluated light and radiation field congruence and the field size dimensions were compared with a film measurement. The dimensions of the light field was determined by subtracting the total cross and in‐plane light and radiation field deviations from the measured length of the radiation field in *X* and *Y*. The light field size was compared to the absolute field size, as defined by the recorded digital collimator jaws.

The films were analyzed via visible inspection and considered the “gold standard” to compare the EPI results. The light field was defined as the distance between the two parallel marks drawn on the film. Similar to the EPIs, the light field size was compared with the absolute field size.

This procedure was repeated on three machines to determine whether the linac‐EPID combination influenced the results. Weekly portal images were acquired on a Varian Clinac 2100C without a multileaf collimator system and a Varian Clinac 21EX equipped with a Millennium MLC‐120, for a two month period, and a Varian Clinic 2100C equipped with a Mk 2 MLC‐80 for a total of eight months. The sensitivity of the EPID to detect small changes in the radiation field was also tested by intentionally applying a 1‐mm shift in *X* and *Y* for low and high‐energy photon beams.

## RESULTS

Three observers independently measured light and radiation field congruence with both film and EPIs for one of the three linacs under study. The results were recorded and monitored for a period ranging from 2–8 months. A total of 67 EPIs and 67 films were analyzed. At the completion of the study, the systematic and random errors for each method were calculated and compared. The deviations along each side of the projected field are summarized in Table I for each machine. In addition, results from opposing field sides were combined to yield the overall cross (Δ*X*) and in‐plane (Δ*Y*) deviations. Based on these results, no appreciable difference was observed in light and radiation field congruence between the conventional and the EPID‐based technique discussed in this study (p–value>0.2 for Δ*X* and Δ*Y* for all machines except the Δ*Y* for machine *C*, 0.15<p–value<0.2). A systematic error of less than or equal to 0.6 mm has been calculated for the cross and in‐plane discrepancies. In addition, the low‐energy (6 MV) and high‐energy (10 or 18 MV) results compare well (p–value>0.2), suggesting that light radiation field congruence is independent of the energy of the incident photons.

**Table I:** The systematic and random errors for light and radiation field congruence based on electronic portal images and films measurements for A, the Varian Clinac 2100C without a MLC system, B, the Varian Clinac 2100C equipped with an MLC‐80 and C the Varian 21EX equiped with an MLC‐120.

		Top (mm)	Bottom (mm)	Left (mm)	Right (mm)	ΔX (mm)	ΔY (mm)
A	EPI 6MV	–0.2+/–0.5	–0.1+/–0.2	0.1+/–0.6	0.1+/–0.6	0.2+/–0.8	–0.4+/–0.6
	Film 6MV	–0.2+/–0.4	0.2+/–0.3	0+/–0.3	0.2+/–0.4	0.2+/–0.6	–0.1+/–0.6
	EPI 10 MV	0.2+/–0.4	–0.2+/–0.4	0.1+/–0.6	0.3+/–0.4	0.4+/–0.7	0+/–0.8
	Film 10 MV	0+/–0.3	−0.2+/−0.5	0+/–0.3	0.3+/–0.4	0.3+/–0.5	–0.1+/–0.3
B	EPI 6MV	0.3+/–0.6	0.2+/–0.5	0.2+/–0.4	0.1+/–0.4	0.3+/–0.7	0.1+/–0.8
	Film 6MV	0+/–0.3	0.1+/–0.3	0.1+/–0.4	0.3+/–0.5	0.3+/–0.7	0.1+/–0.5
	EPI 10MV	0.1+/–0.6	0.1+/–0.7	0.2+/–0.4	0.2+/–0.5	0.4+/–0.7	0.3+/–0.7
	Film 10MV	0+/–0.4	0.3+/–0.4	0.1+/–0.3	0.2+/–0.5	0.3+/–0.6	0.3+/–0.6
C	EPI 6MV	0.6+/–0.5	0.5+/–0.6	0.5+/–0.5	0.2+/–0.6	0.3+/–0.7	0.1+/–0.9
	Film 6MV	0.2+/–0.4	0.4+/–0.5	0.2+/–0.4	0.2+/–0.4	0.3+/–0.7	–0.3+/–0.8
	EPI 18MV	0.5+/–0.6	0.1+/–0.7	0.3+/–0.4	0.1+/–0.5	0.2+/–0.5	0.6+/–0.8
	Film 18MV	0.2+/–0.4	0.1+/–0.4	0.1+/–0.3	0+/–0.5	0.1+/–0.4	0+/–0.7

A comparison of the absolute field size as defined by the digital collimator jaw settings and the light field detected on the films and EPIs is presented in Table II. Based on these results, the size of the detected light field differs depending on the QA technique utilized. The film measurements are in better agreement with the digital jaw settings, yielding a difference of <1.4 mm, whereas the EPIs over‐estimated the size of the light field by up to 2.5 mm. The magnitude of the systematic error for the low and high‐energy *x*‐ray beams, for a given QA procedure, is consistent (p‐value>0.2), again suggesting that the results are independent of the energy of the irradiating beam.

The EPID was also found to be sensitive to small changes made to the size of the radiation field. Portal images were acquired before and after an intentional 1‐mm shift in *X* and *Y* was applied to the collimator jaws. After measuring and comparing the detected radiation field size, a 1‐mm difference was observed in *X* and *Y* between the two portal images.

**Table II:** A summary of the difference between the digital collimator setting for an acquired portal image and the light field defined on film and EPIs. The ΔX and ΔY values for films and EPIs are compared and the respective p‐values are listed.

		Δ*X* (mm)	*p*‐value	Δ*Y* (mm)	*p*‐value
Machine A	EPI 6MV	2.0+/–0.8		2.5+/–0.6	
	Film 6MV	0.5+/–0.6	<0.001, >0.0005	1.1+/–0.4	<0.0005, >0.0001
	EPI 10MV	1.8+/–0.7		2.3+/–0.7	
	Film 10MV	0.6+/–0.7	<0.005, >0.001	1.4+/–0.4	<0.01, >0.005
Machine B	EPI 6MV	1.1+/–0.1		–0.2+/–1.0	
	Film 6MV	0.4+/–0.8	<0.05, >0.02	0.7+/–1.0	<0.2, >0.1
	EPI 10MV	1.2+/–0.8		0+/–0.7	
	Film 10MV	0.5+/–0.9	<0.05, >0.02	–0.5+/–1.1	>0.2
Machine C	EPI 6MV	1.1+/–0.4		0.8+/–0.8	>0.2
	Film 6MV	0+/–0.3	<0.0005, >0.0001	0.3+/–1.1	
	EPI 18MV	1.3+/–0.5		0.5+/–0.8	>0.2
	Film 18MV	0.1+/–0.5	<0.001, >0.0005	0.1+/–1.1	

## DISCUSSION

The EPID‐based quality assurance technique discussed in this study is a relatively fast and reliable method for checking light radiation congruence. The total time to acquire and analyze a port is on the order of 1.5 min. The results presented in Table I suggest that the EPID‐based technique is comparable to film, reporting discrepancies on the order of a fraction of a millimeter (p–value>0.2 for all three machines). As the diamond template easily mounts into a block tray holder, it may be used to test light radiation field congruence at any gantry angle. Factoring in the time required to setup and process the film, and the expense of the films and processing chemicals, substituting EPIs for films will prove to be more labor and cost‐effective at facilities already equipped with EPIDs.

A difference was observed in the measured size of the light field between films and EPIs. The size of the light field measured with film was comparable to the digital collimator settings. At most, a 1.4 mm discrepancy was observed in‐plane (Δ*Y*) for machine *A*. However, the EPID measurements were offset by up to an additional 1.5 mm. This is in agreement with Dunscombe *et al*.^5^ study using a camera‐based EPID, although the magnitude of their difference is considerably smaller, on the order of 0.5 mm. This difference may be attributed to vertical misalignments of the imager due to detector sag or positional calibration. A one‐centimeter tolerance is allowed for the vertical position of the imager. Thus, by moving the imager to the 160‐cm pre‐programmed position, it may actually sit between 159–161 cm. As the images are scaled back to isocenter, 100 cm source detector distance, a vertical offset of 1 cm may result in up to a 1.5 mm discrepancy in the detected *X* and *Y* dimensions. This effect would not be observed when testing light and radiation field congruence because the jaws are set to cast shadows congruent with the vertexes of the diamond template on the surface of the detector and so both light and radiation fields are magnified by an equivalent factor. However, inaccurate positioning of the EPID becomes apparent with analysis of absolute field size.

## CONCLUSION

In this study, the use of an EPID for light and radiation field congruence tests was examined. The results indicate that the EPID‐based procedure is comparable to film, the “gold standard.” Both the conventional and EPID‐based light and radiation field congruence techniques have been shown to be sensitive to below the allowed 2 mm or 1% discrepancy on each side of the square field.^7^ Considering the additional time to setup and process film, the EPID‐based technique is a fast and reliable alternative.

A difference has been observed in the measured dimensions of the light field between film and EPIs. When comparing the field size to the digital collimator settings, the film measurements are in better agreement than the EPIs. This discrepancy may be attributed to a vertical misalignment of the detector, which can adversely affect the scaling of the acquired portal image. As suggested by Dunscombe *et al*.,^5^ if the EPID is to be used to determine absolute field dimensions, it will be necessary to properly calibrate the position of the detector. With the growing desire to implement EPID for IMRT quality assurance, integrating EPIDs into conventional radiation therapy QA is an important first step.

## ACKNOWLEDGMENT

This work was supported in part by Varian Medical Systems.
